# Ocular manifestations in a patient with Dandy-Walker malformation: A case report

**DOI:** 10.1016/j.radcr.2021.12.027

**Published:** 2021-12-31

**Authors:** Qirat Qurban, Zeeshan Kamil, Sameer Saleem Tebha, Zain Ali Zaidi, Maahirah Said, Samar Fatima Zehra, Sajjad Ali, Sehrish Sethar

**Affiliations:** a Department of Ophthalmology, Jinnah Medical and Dental College, Karachi, Pakistan; b Department of Neurosurgery and Neurology , Jinnah Medical and Dental College, Karachi, Pakistan; c Department of Medicine and Allied, Jinnah Medical and Dental College, Karachi, Pakistan; d Department of Medicine, Ziauddin University, Karachi, Pakistan; e Department of Medicine, Jinnah Medical and Dental College, Karachi, Pakistan; f Department of Medicine, Ziauddin Medical University, Karachi, Pakistan; g Department of Radiology, Jinnah Medical and Dental College, Karachi, Pakistan

**Keywords:** Dandy walker Malformation, Posterior fossa, Vermis hypoplasia, Enlarged fourth ventricle, Ocular manifestation, Buphthalmos, Vitreous haemorrhage

## Abstract

We present a unique case of a ten-month-old boy with a protruding left globe and vitreous haemorrhaging, and later being diagnosed as a case of a dandy-walker syndrome (DWS) with buphthalmos and vitreous haemorrhage. Treatment is depending on the symptoms reported, thus close monitoring and a multidisciplinary approach are essential. We would like to recommend that even if there are no cardinal symptoms of DWS, paediatric patients with ocular signs should have Dandy walker Malformation (DWM) considered as a differential diagnosis.

## Introduction

Dandy-Walker Syndrome (DWS) is a congenital malformation usually manifesting as enlargement of the posterior cranial fossa along with cystic expansion of the fourth ventricle and hypoplastic cerebellar vermis [1]. Out of several variants, Dandy-walker malformation (DWM) includes all characteristic radiological findings of vermis hypoplasia, posterior fossa enlargement and ventricular dilation. However, other variants include the Dandy-walker variant (DWV), with no posterior fossa involvement and Mega-cisterna magna that manifests as enlarged cistern magna with normal ventricles and vermis [1].

The incidence of this rare disorder ranges from 1 in 25,000 to 1 in 35,000 live births, with findings indicating a probable link to chromosomal anomalies. [2] Suton was the first to describe the illness in 1887, and Benda was the first to give it the label DWS in 1955 [3]. Vomiting, convulsions, irritability, interrupted sleep, and uncoordinated muscle movements are among the first symptoms of DWS [1]. It presents clinically as growth and mental retardation, brachycephaly [1] (an unusually enlarged nose), macrocephaly (an increase in the space between the palpebral fissures) [4] , and a range of ocular signs and symptoms such as myopia, nystagmus, strabismus, microphthalmia, and neuropathies [5]. Despite the large variety of clinical characteristics and symptoms, no single finding has been proven to be diagnostic of this condition, hence the diagnosis is usually determined based on firm radiological evidence [1].

Here, the authors discuss a case of a ten-month-old kid who presented with a bulging left globe and vitreous haemorrhage and was later diagnosed as DWS with buphthalmos and vitreous haemorrhage. To our knowledge, no case of DWS with ocular manifestations of buphthalmos and vitreous haemorrhage has ever been recorded in previous literature.

## Case presentation

**Fig. 1:**
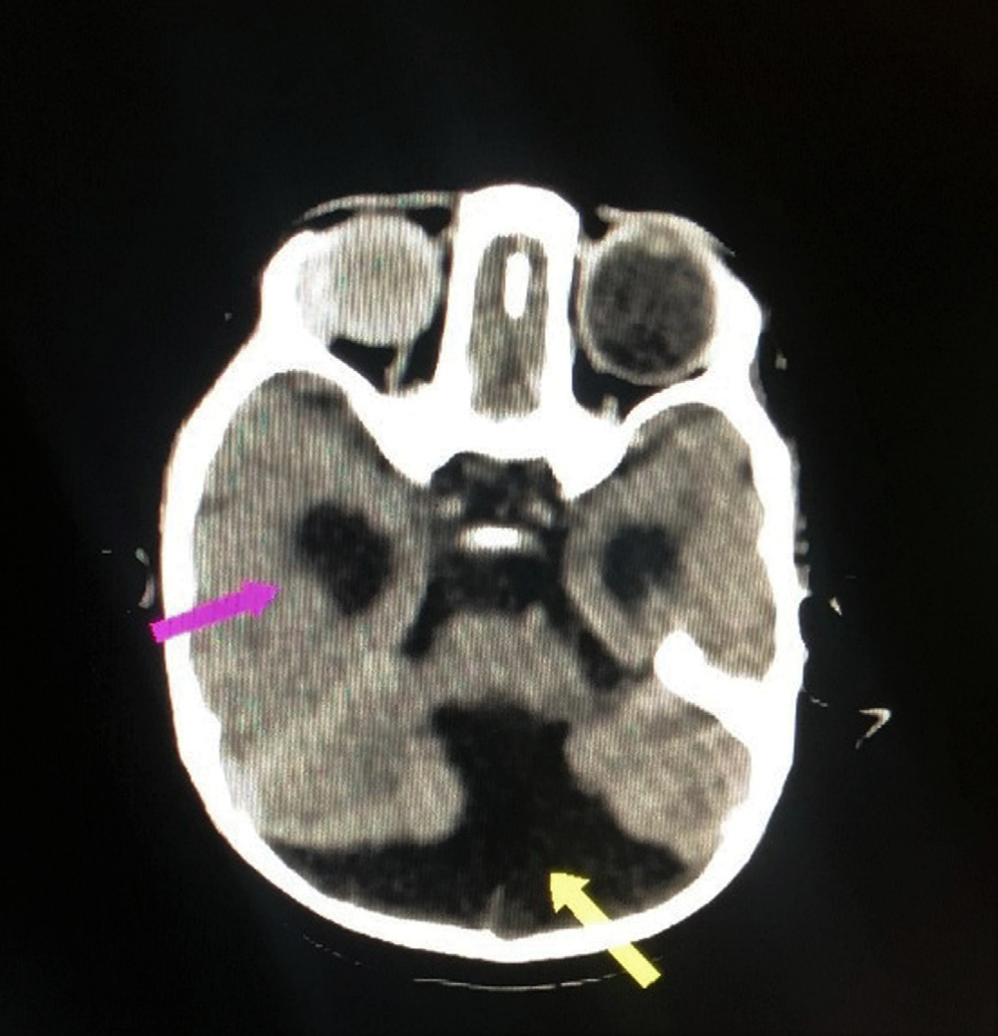
**–** Hyperattenuation in the vitreous chamber on the right side representing vitreous haemorrhaging

**Fig. 2:**
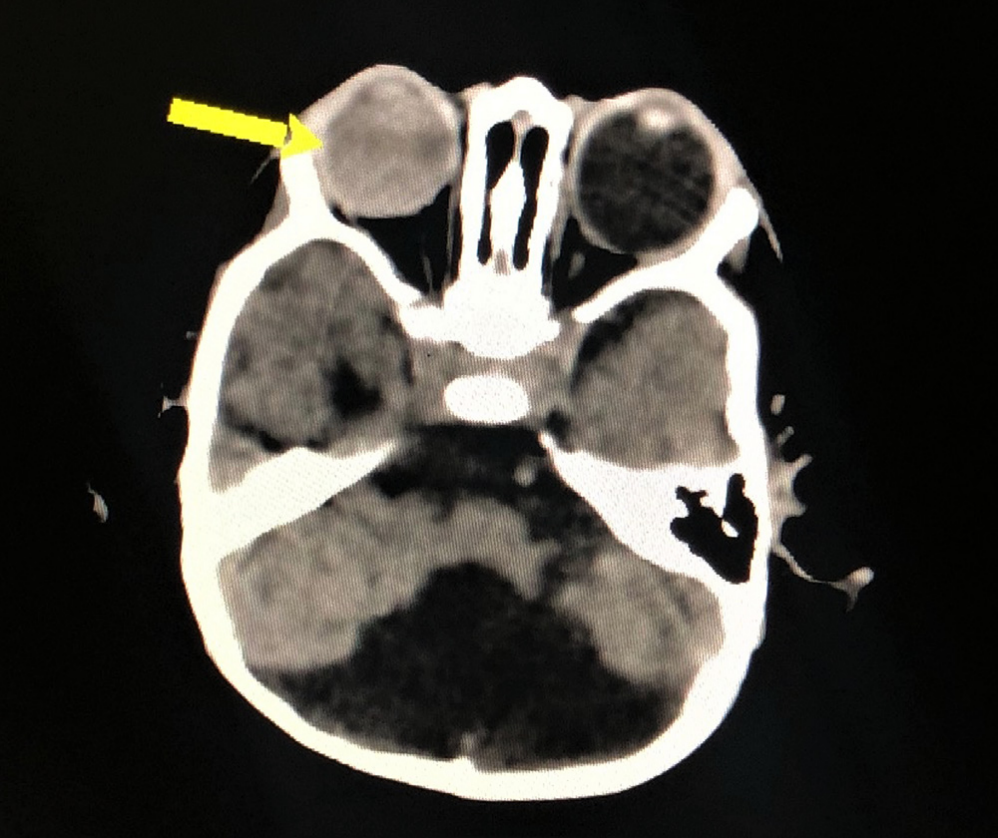
**–** There is diffused enlargement of bilateral eye globes noted measuring 2.4 cm on the right and 2.6 cm on the left in the anteroposterior diameter

**Fig. 3:**
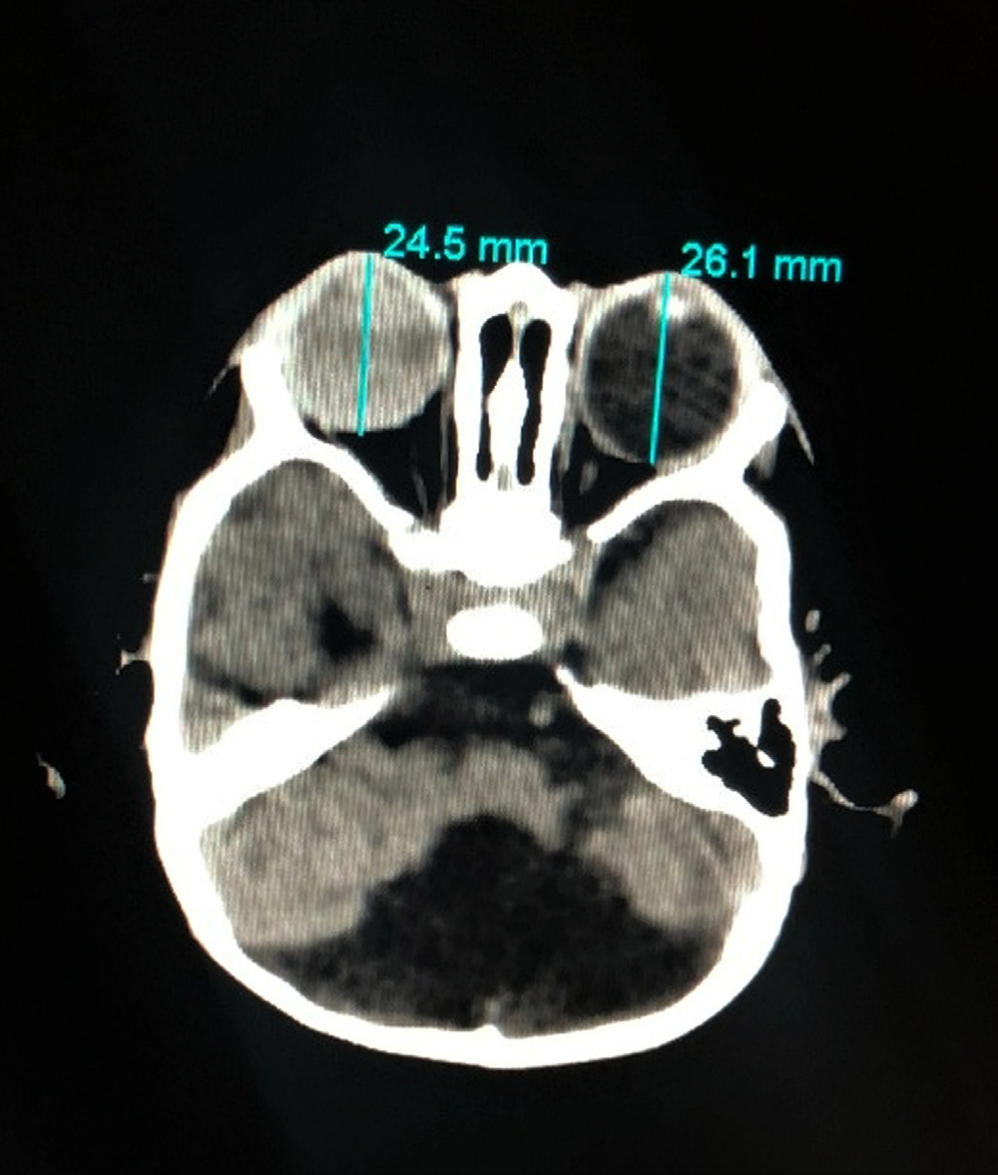
**–** (yellow arrow): Axial CT image shows midline open communication of the fourth ventricle with a large cystic posterior fossa. The cerebellar hemisphere is hypoplastic representing dandy walker malformation (purple arrow): The temporal horns of the lateral ventricle are also dilated

A 10-month-old boy from Nawabshah, Pakistan, a result of consanguineous marriage (marriage between first blood relatives, i.e. cousins), reported to the Paediatric department with the major complaint of conspicuous and projecting globes with watery discharge during the previous 6 months. The child was agitated, had disturbed sleep, uncoordinated muscular movement, and a lack of interest and concentration. On antenatal history, the mother, G3P0 notified of not taking any medication or supplements for folic acid, vitamins or iron and did not undergo any radiological investigation and had no symptoms of pyrexia, convulsions or vaginal bleeding throughout her pregnancy. She underwent full-term spontaneous vaginal delivery with no complications to the mother or the newborn. Following the pediatric evaluation at the time of birth, the patient weighed 2.5kgs (2.9 percentile) and his occipitofrontal circumference was 32.1 cm (11.5 percantile). The patient had developed bilateral protrusion and enlargement of the globes over the previous 6 months and was immediately referred for a computed tomography scan (CT) without contrast, which revealed cystic enlargement of the fourth ventricle with an enlarged posterior fossa and hypoplasia of the cerebellar hemisphere (Fig. 3), assisting in the diagnosis of DWS. The patient also exhibited bilateral buphthalmos (Fig. 2) and ocular haemorrhage (Fig. 1). Other causes of buphthalmos, such as aniridia, sclerocornea, and sources of vitreous haemorrhages, such as eye trauma, were ruled out after a thorough history and examination. This highly unusual finding of ocular symptoms in a DWS patient is the first of its kind. The patient was also advised to get an A and B scan to further evaluate the ocular signs, but the non-affordability was an issue. Because there is no definite cure for DWS and we can only treat the underlying symptoms, the patient's family was offered surgical treatment for buphthalmos but rejected and chose not to pursue treatment. Before the construction of this case report, the patient's parents were taken into confidence, informed consent was obtained from them, and they are kept up to date on the status of this manuscript frequently.

## Discussion

DWS is a rare congenital brain abnormality that is hypothesized to be caused by a combination of environmental and genetic causes. Prenatal exposure to some viruses and medicines, such as alcohol and other teratogenic substances, may be environmental influences. [6] The disorder expresses itself as a range of signs and symptoms, including ocular involvement; nevertheless, the link of DWS with buphthalmos and vitreous haemorrhage is yet novel in the fields of neurology and ophthalmology.

Buphthalmos is an uncommon ophthalmologic condition with an incidence of 1 in 30,000 live births, characterized by apparent enlargement of the eye that might be congenital or develop soon after birth. It can be a result of congenital glaucoma, and can also be associated with conditions like aniridia, neurofibromatosis and Sturge-weber syndrome which all cause increased Intra-ocular pressure. [7] However, no case has ever been reported of DWS with ocular manifestation of buphthalmos. Similarly, no case of DWS along with the diagnosis of vitreous haemorrhage has been reported in previous literature. This makes our case extremely unconventional from the usual presentation, as we diagnosed a case of DWS along with the clinical finding of bilateral buphthalmos and vitreous haemorrhage. This brings a new addition to the variety of other signs and symptoms of DWS and would aid in diagnosis in the near future.

For our literature search, we reviewed PubMed and we were unable to find any similar case through our literature search we used the search term “Dandy-Walker Malformation” OR “Syndrome” OR “Anomaly” AND “Occular Manifestations” OR “Eye Anomalies” OR “Ophthalmic Manifestations” and we did not use any filters.

One could argue that these presentations are coincidental, but in the authors' clinical opinion, these three rare anomalies should present in a manner similar to ours, where buphthalmos, which almost always develops at or very soon after birth if congenital, and vitreous haemorrhaging most commonly have a traumatic a etiology in children, whereas this does not appear to be the case in our cases, making it difficult to consider these presentations as coincidental.

## Conclusion

DWS may be associated with rare ocular involvement of buphthalmos and even vitreous haemorrhage as in our case. The imaging indicates a clear presentation of the DWM with bilateral buphthalmos and vitreous haemorrhage on ocular investigations. The clinical presentation along with extensive history and radiological examinations confirms the diagnosis. Close monitoring along with a multidisciplinary approach is important, as treatment is dependent upon symptoms presented. Through our report, we would like to suggest that pediatrics patients with ocular manifestations should have DWM as a differential diagnosis even without any cardinal symptoms of DWS. Although we were unable to perform an A and B scan, we were able to exclude the major and most causes of these ocular manifestations through detailed history and examination.

## Authors’ contributions

Sameer Saleem Tebha and Zain Ali Zaidi: Wrote the initial draft of the manuscript, did a detailed literature review; Qirat Qurban and Zeeshan Kamil: Wrote the discussion, edited the case presentation, and followed the patient along with taking the consent; Maahirah Said: Develop then list of differentials and helped in the clinical exclusion of them; Sehrish Sethar: Identified the rare finding, performed the radiographic examination, and interpreted the finding; Syeda Samar Fatima Zehra Zaidi and Sajjad Ali: Did the final review and proofread the manuscript. All authors approved the final version for publication.
